# Hyperfunctioning Solid/Trabecular Follicular Carcinoma of the Thyroid Gland

**DOI:** 10.1155/2010/635984

**Published:** 2010-08-16

**Authors:** Luca Giovanella, Fabrizio Fasolini, Sergio Suriano, Luca Mazzucchelli

**Affiliations:** ^1^Department of Nuclear Medicine and PET-CT Centre, Oncology Institute of Southern Switzerland, 6500 Bellinzona, Switzerland; ^2^Department of Surgery, Ente Ospedaliero Cantonale, Ospedale Regionale di Mendrisio, 6850 Mendrisio, Switzerland; ^3^Department of Clinical Pathology, Cantonal Institute of Pathology, 6600 Locarno, Switzerland

## Abstract

A 68-year-old woman with solid/trabecular follicular thyroid carcinoma inside of an autonomously functioning thyroid nodule is described in this paper. The patient was referred to our clinic for swelling of the neck and an increased pulse rate. Ultrasonography showed a slightly hypoechoic nodule in the right lobe of the thyroid. Despite suppressed TSH levels, the ^99m^Tc-pertechnetate scan showed a hot area corresponding to the nodule with a suppressed uptake in the remaining thyroid tissue. Histopathological examination of the nodule revealed a solid/trabecular follicular thyroid carcinoma. To the best of our knowledge, this is the first case of hyperfunctioning follicular solid/trabecular carcinoma reported in the literature. Even if a hyperfunctioning thyroid carcinoma is an extremely rare malignancy, careful management is recommended so that a malignancy will not be overlooked in the hot thyroid nodules.

## 1. Introduction

Hyperthyroidism due to thyroid carcinoma is an extremely rare phenomenon. It is commonly believed that the diagnosis of a solitary autonomously functioning thyroid nodule (AFTN)—a solitary “hot” nodule in radionuclide imaging—can almost always rule out malignancy in the nodule [1]. In this paper, we present the rare case of follicular carcinoma manifesting as an AFTN.

## 2. Case Report

**Figure 1:**
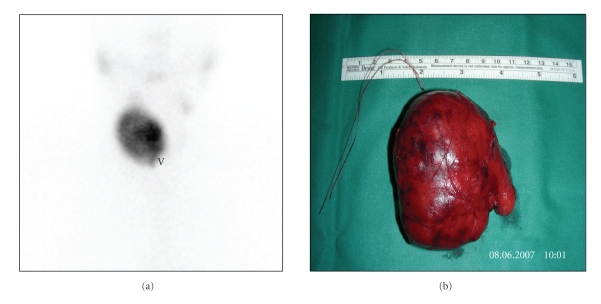
^99m^Tc scan: hot thyroid nodule in the right thyroid lobe with suppressed extranodular thyroid tissues (a). Surgical specimen from right lobectomy and isthmectomy (b).

**Figure 2:**
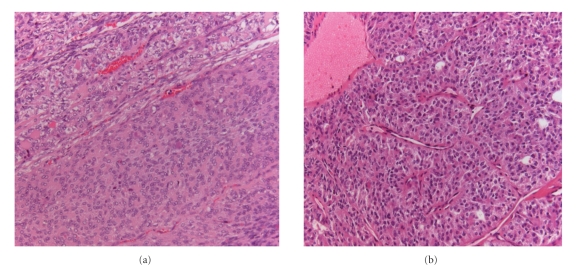
Hematoxylin/eosin histological stains: follicular carcinoma with solid (a) and solid-trabecular (b) features.

A 68-year- old female, affected by a long-standing asymptomatic normally functioning nodule in the right lobe of the thyroid, developed symptoms of neck swelling and palpitations. The patient presented a resting pulse rate of 108 and blood pressure of 145/90 mmHg. A large painless, well-defined, hard nodule was palpable in the right lobe of the thyroid; the left lobe was normal, and there were no cervical lymphadenopathies. Ultrasonography (US) of the thyroid revealed a large and slightly hypoechoic nodule (diameters: 33 × 38 × 53 mm). Thyroid function tests showed elevated free triiodothyronine (fT3) of 7.60 pmol/L (reference range 2.30–6.30 pmol/L) and undetectable thyroid-stimulating hormone (TSH) of 0.006 mIU/L (normal 0.4–4.0 mIU/L). The free thyroxin (fT4) was normal at 11.4 pmol/L (reference range 7.5–21.1 pmol/L), and both thyroperoxidase and thyrotropin-receptor autoantibodies were negative (<60 UI/mL and <1 U/L, resp.). A ^99m^Tc-perthechnetate scan demonstrated a large hot area with inhomogeneous uptake and no cold areas inside corresponding to the nodule, with a suppressed uptake in the remaining thyroid tissue (Figure 1(a)). Six and 24 hours radioiodine uptake (RAIU) values were 10% (normal: 5–15%) and 32% (normal: 10–35%), respectively. The nodule was considered an AFTN, but radioiodine treatment (that is the first-line treatment for AFTN in our centre) was ruled-out based on the symptoms (i.e., neck swelling) and the volume of the nodule. Right loboisthmectomy was performed (Figure 1(b)) and the histological examination revealed a follicular carcinoma with solid and trabecular parts and focal signs of angioinvasivity (Figures 2(a), (b)). The surrounding thyroid tissue showed a follicular architecture with no signs of tumour infiltration or spreading. Since the patient declined further surgery, a radioiodine ablation was directly performed by administering ^131^I (2.5 GBq). Serum thyroglobulin was 9.4 ng/mL before ^131^I treatment, with a corresponding TSH level of 36 mUI/L. Six months after thyroid ablation, a ^131^I whole-body scanning after recombinant human TSH administration was negative with a corresponding undetectable serum Tg (i.e., <0.2 ng/mL). Further followup by clinical examination, including neck US and Tg measurement, every 6 months, is negative up to now (3.4 years follow-up).

## 3. Discussion

Our patient presented with a palpable thyroid nodule and hyperthyroidism with the absence of TRAb and TPOAb. The nodule was proved to be functionally autonomous by ^99m^Tc-pertechnetate imaging and RAIU. However, a follicular solid/trabecular carcinoma was finally proved by histological examination. Hyperthyroidism due to thyroid carcinoma is a rare, but well-recognized phenomenon. This situation has been generally described as resulting from excessive production of thyroid hormone by extensive functioning metastases, usually from follicular carcinoma [2, 3]. The incidence of thyroid carcinoma in a hot nodule is reported to be very low by most authors [4–6], but the incidence is somewhat higher in other retrospective studies [7, 8]. Actually, thyroid carcinoma in a hot nodule has been described in numerous case reports prior to ours. However, unlike our case, most of these cases show a cold area within a hot nodule, indicating that the thyroid carcinoma itself did not produce thyroid hormone [9]. Women are far more often affected than men, but no significant peak with regard to age was noted [10]. Interestingly, the histological features of these tumors correspond in principle to the papillary carcinoma, as opposed to the metastatic functioning carcinomas, essentially being of follicular type [11–15]. Classical follicular histology is described in the few reported cases of hyperfunctioning follicular carcinoma while only one case with a clear-cell variant histotype is described [16–18]. To the best of our knowledge, we are the first to report a case of hyperfunctioning aggressive follicular carcinoma with solid and trabecular features. This case underlines the clinical importance of predicting the incidence of malignancy in hot thyroid nodules. However, reports in the literature indicate significant difficulty in determining the risk that AFTN will undergo malignant degeneration. Some clinical findings set forth the risk factors for malignancy in thyroid nodules: age <20 or >60 years, male sex, the family history of differentiated or medullary thyroid carcinoma or of familial adenomatous polyposis (Gardner's syndrome), past history of head and neck radiation, rapid tumor growth, irregular outline, fixation to adjacent structures, and symptoms of tumor invasion [1, 15, 19]. In actual practicei, however, few patients have these symptoms, and most nodules are nearly asymptomatic [1]. The classical benign AFTN presents itself as a smooth, well-defined, round or ovoid mass that moves freely and occurs in patients aged 40 or over with a history of long-standing and slowly expanding mass in the neck [19]. The US pattern, as well as the vascular signals in power or color-Doppler samplings, is largely overlapped in malignant nodules and AFTN, as occurred in our patient [1]. An incomplete suppression of radionuclide uptake in extranodular thyroid tissues was reported as a risk factor of malignancy, but this did not occur in our patient [20]. Differentiating a benign follicular adenoma from a malignant follicular carcinoma is challenging by cytology, and a thyroid scan is advocated in these cases, considering functioning nodules as being benign [1, 21]. Hot nodules outside the thyroid can be helpful in diagnosis of malignancy in the case of metastatic thyroid carcinoma, but this is rare in practice [22]. In our patient, surgical treatment was preferred to radioiodine ablation considering her symptoms and the nodule's size. However, ^131^I could be administered in patients with similar clinical presentation; a progressive increase in the nodule size after ^131^I treatment was signalled as a suspicious sign for malignancy in these cases and should be promptly evaluated [23]. A possible reason for why thyroid carcinomas occasionally produce excessive hormone without extensive metastases could be due to gene mutations reported to occur in AFTN, including mutations of G protein *α* chain gene and TSH-receptor gene with associated elevated intracellular cAMP [19]. By contrast, Bourasseau et al. denied TSH-receptor and G protein *α* chain gene mutation in hyperfunctioning thyroid carcinoma [24]. Consequently, further studies are needed to clarify this issue. The clinical course of a nonmetastatic hyperfunctioning thyroid carcinoma depends on its histological features and on the patients' age and tumor stage at the time of diagnosis [25]. It seems that the prognosis of metastatic follicular carcinoma does not differ with the presence or absence of hyperthyroidism [15, 16, 26]. However, the prognosis of nonmetastatic hyperfunctioning papillary and follicular thyroid carcinoma was not fully described in the literature. In conclusion, our case showed that an aggressive follicular thyroid carcinoma with solid/trabecular features without metastases could produce hyperthyroidism, suggesting that malignancy (even if very rare) cannot always be excluded in a hot thyroid nodule. Consequently, careful management is recommended so that malignancy is not overlooked in the scintigraphically hyperfunctioning nodules.

## References

[1] (2006). American Association of Clinical Endocrinologists and Associazione Medici Endocrinologi medical guidelines for clinical practice for the diagnosis and management of thyroid nodules. *Endocrine Practice*.

[2] Kasagi K, Takeuchi R, Miyamoto S (1994). Metastatic thyroid cancer presenting as thyrotoxicosis: report of three cases. *Clinical Endocrinology*.

[3] Davis TF, Larsen PR, Larsen PR, Kronenberg HM, Melmed S, Polonsky KS (2003). Thyrotoxicosis. *Williams of Textbook of Endocrinology*.

[4] Harach HR, Sánchez SS, Williams ED (2002). Pathology of the autonomously functioning (hot) thyroid nodule. *Annals of Diagnostic Pathology*.

[5] Chao T-C, Lin J-D, Jeng L-B, Chen M-F (1999). Thyroid cancer with concurrent hyperthyroidism. *Archives of Surgery*.

[6] Rieger R, Pimpl W, Money S, Rettenbacher L, Galvan G (1989). Hyperthyroidism and concurrent thyroid malignancies. *Surgery*.

[7] Smith M, McHenry C, Jarosz H, Lawrence AM, Paloyan E (1988). Carcinoma of the thyroid in patients with autonomous nodules. *American Surgeon*.

[8] Mizukami Y, Michigishi T, Nonomura A (1994). Autonomously functioning (hot) nodule of the thyroid gland: a clinical and histopathologic study of 17 cases. *American Journal of Clinical Pathology*.

[9] Lupi A, Orsolon P, Cerisara D, Deantoni Migliorati G, Vianello-Dri A (2002). "Hot" carcinoma of the thyroid. Case reports and comments on the literature. *Minerva Endocrinologica*.

[10] Yaturu S, Fowler MR (2002). Differentiated thyroid carcinoma with functional autonomy. *Endocrine Practice*.

[11] Baumann K, Weitzel M, Burgi H (1979). Hormone-producing thyroid carcinoma with hyperthyroidism. Analysis of 6 cases and review of the literature. *Schweiz Med Wochenschr*.

[12] Fukata S, Tamai H, Matsubayashi S (1987). Thyroid carcinoma and hot nodule. *European Journal of Nuclear Medicine*.

[13] Appetecchia M, Ducci M (1998). Hyperfunctioning differentiated thyroid carcinoma. *Journal of Endocrinological Investigation*.

[14] Hayata M, Kamei T, Okayasu N (2003). Functional papillary carcinoma of the thyroid occurred in the Graves’ disease. *Clinical Endocrinology*.

[15] Paul SJ, Sisson JC (1990). Thyrotoxicosis caused by thyroid cancer. *Endocrinology and Metabolism Clinics of North America*.

[16] Tsuchiya A, Nemoto T, Nomizu T, Sato H, Watanabe I, Abe R (1987). Follicular carcinoma in an autonomously functioning thyroid nodule. *Gan no Rinsho*.

[17] Niepomniszcze H, Suárez H, Pitoia F (2006). Follicular carcinoma presenting as autonomous functioning thyroid nodule and containing an activating mutation of the *TSH Receptor* (T620I) and a mutation of the *Ki-RAS* (G12C) genes. *Thyroid*.

[18] Schneider PW, Meier DA, Balon H (2000). A clear cell variant of follicular carcinoma presenting as an autonomously functioning thyroid nodule. *Thyroid*.

[19] Mazzaferri EL (1993). Management of a solitary thyroid nodule. *The New England Journal of Medicine*.

[20] De Rosa G, Testa A, Maurizi M (1990). Thyroid carcinoma mimicking a toxic adenoma. *European Journal of Nuclear Medicine*.

[21] Mann K (1998). Evaluation of risk in autonomously functioning thyroid nodules. *Experimental and Clinical Endocrinology and Diabetes*.

[22] Yamamoto Y, Nishiyama Y, Ono Y (1999). Accumulation of technetium-99m pertechnetate in a patient with metastases of thyroid carcinoma. *Annals of Nuclear Medicine*.

[23] Uludag M, Yetkin G, Citgez B, Isgor A, Basak T (2008). Autonomously functioning thyroid nodule treated with radioactive iodine and later diagnosed as papillary thyroid cancer. *Hormones*.

[24] Bourasseau I, Savagner F, Rodien P (2000). No evidence of thyrotropin receptor and G(s*α*) gene mutation in high iodine uptake thyroid carcinoma. *Thyroid*.

[25] Mazzaferri EL, Braverman LE, Utiger RD (1991). Carcinoma of follicular epithelium: radioiodine and other treatment and outcomes. *Werner and Ingbar’s the Thyroid: A Fundamental and Clinical Test*.

[26] Lin J-D, Chao T-C, Hsueh C (2004). Follicular thyroid carcinomas with lung metastases: a 23-year retrospective study. *Endocrine Journal*.

